# Missing heritability and where to find it

**DOI:** 10.1186/s13059-017-1227-x

**Published:** 2017-05-11

**Authors:** Santhosh Girirajan

**Affiliations:** 1 Department of Biochemistry and Molecular Biology, 205A Huck Life Sciences Building, Pennsylvania State University, University Park, PA 16802 USA; 2 Department of Anthropology, 205A Huck Life Sciences Building, Pennsylvania State University, University Park, PA 16802 USA

## Abstract

A report on the 11th Genomics of Rare Disease meeting held at the Wellcome Genome Campus, Hinxton, Cambridge, UK, 5–7 April, 2017.

## Still plenty of real estate in the genome

High-throughput and affordable genomic technologies have spawned several large-scale genetic studies on human disease populations. What might have been disparate disciplines a decade ago, including clinical medicine, biology, and computer science, are now converging with the goal of understanding the complex basis of human disease. The 11th Genomics of Rare Disease conference focused not only on the often-missed components of genetic analysis, but also featured topics related to key clinical phenotypes and molecular pathways of disease, technologies for high-throughput functional studies, and cellular and animal models. In this report, I summarize my highlights of the conference and also provide my personal perspectives of the meeting. I would like to emphasize that any omissions in this report are not based on quality, but on the content that can be most coherently presented in limited space.

While causative genes for a vast majority of Mendelian disorders have been discovered, large-scale exome and whole-genome sequencing studies that are focused on protein-coding regions of the genome have accounted for only about 40% of the genetic basis of complex rare disorders. This problem of missing heritability has been haunting geneticists for about a decade, as the initial promises and high expectations from genomic technologies did not match with the reality of complex genetics. Technological advances have mostly dictated our knowledge of the genome and its assessment for causal traits, and recent developments in genomic analysis have moved our focus beyond the protein-coding regions of the genome. Several speakers dared to talk about the role of the non-coding genome, splice sites, and common variants in developmental disorders, a “laundry list” of elements drawn from a classic genetics textbook that have been neglected. For example, Matthew Hurles (Wellcome Trust Sanger Institute, UK), reporting on data from more than 8000 individuals from the Deciphering Developmental Disorders project, presented evidence for enrichment of de novo variants within highly conserved non-coding regions that are active in fetal brains. However, he also noted that only about 2% of these variants could be deemed as pathogenic, and it would require at least 100,000 trios to gain power to detect such variants reliably.

Mari Niemi (Wellcome Trust Sanger Institute, UK) also used the Deciphering Developmental Disorders cohort, taking advantage of the large sample size to assess the role of common variation in developmental disorders. While no SNPs achieved genome-wide significance, co-heritability analysis showed a negative correlation between risk for the disorder and years of schooling. Hilary Martin (Wellcome Trust Sanger Institute, UK) dug deeper to show that recessive causes of developmental disorders due to biallelic mutations probably account for a very small fraction of individuals who do not have a diagnosis with exomes, and Joanna Kaplanis (Wellcome Trust Sanger Institute, UK) described how multiple nucleotide variants can occur in the same exon, causing a more severe phenotype even though they are often considered as independent mutations.

## Looking beyond the “exomic lamppost”

It was refreshing to see studies that took genetic diagnosis further by looking at RNA sequencing. While many studies of complex disease focus on either exome sequencing or transcriptome studies in unraveling the genetic complexity of disease, combining both types of data in the same study will provide a more comprehensive view of disease. Beryl Cummings (Broad Institute, USA) demonstrated that novel splice-altering variants from RNA data increase the overall diagnostic yield of exome-based studies by about 30%. Similarly, Laura Kremer (Helmholtz Zentrum München, Germany) showed that RNA sequencing can identify an additional 15% of clinically relevant mutations in mitochondrial diseases. In addition to identifying genes with varying expression in disease, RNA sequencing can uncover alternative splicing of genes that can affect exome-sequencing results. Jenny Lord (Wellcome Trust Sanger Institute, UK) presented data from population genetic and disease-burden analyses showing an enrichment of de novo mutations and positive signals of purifying selection in non-canonical splice sites. More convincing was that the positive predictive value of pathogenicity of these variants correlated well with the clinical classification of patients.

Genetic mosaicism was another category of phenomena that was discussed extensively, and it is clear that we have more disease-causing variants to discover that are present in specific cell types. Veronica Kinsler (UCL Institute of Child Health, UK) broadened the list of searchable genomic space with her talk on mosaicism. It was fascinating to learn the role of mosaic mutations in cancer and other diseases, and how phenotypes can be modulated by incredible interactions at several tissue interfaces between mutant and non-mutant cells. Marie-Louise Bondeson (Uppsala University Hospital, Sweden) presented an example of how skin phenotypes caused by a germline mutation in *GJB2* were rescued by mosaic mutations at a second site in the same gene. These results revealed only one of the myriad of genetic mechanisms that modulate disease phenotypes. Several areas of the genome are still widely understudied by both researchers and clinicians, especially if they may be causative for complex diseases that do not have a consistent phenotype. Karen Temple (University of Southampton, UK) reminded us that a vast majority of disorders caused by genomic imprinting are missed by routine diagnosis testing, as epigenetics is not part of such testing and we tend to expect classic features in individuals with imprinting disorders which may not always be the case.

## Advancing technologies and methods

Our knowledge of the architecture of the human genome is not yet complete, as complex and repetitive DNA and limitations in sequencing technologies have precluded assembling a complete genome. Such limitations are likely coming to an end with newer sequencing methods that can provide longer and deeper coverage of the genome to identify sequences that were previously hidden from genetic analysis. Mark Chaisson (University of Washington, USA) presented data to show that single molecule sequencing produces reads that are two orders of magnitude longer than standard Illumina reads, allowing for accurate detection of structural variants and repeat elements, thereby resolving genomic complexity, resulting in a better genome assembly. Other novel technologies, such as identifying fetal DNA in maternal blood samples, are becoming more and more prevalent in clinical labs, increasing the accuracy and precision of genetic diagnosis. Rossa Chiu (Chinese University of Hong Kong, PR China) commented that non-invasive prenatal diagnosis has reduced the number of invasive procedures by 30%. She presented non-invasive strategies for prenatal detection of single gene diseases and suggested how biological mechanisms underlying fragmentation of cell-free DNA could be used for improving the diagnosis.

## Going functional

Functional studies of genes uncovered in human genomic studies will help connect genes to phenotypes through a biological pathway, which can then be used for biomarker diagnosis as well as drug targeting. Han Brunner (Radboud University Medical Center, The Netherlands) and Kate Tatton-Brown (Institute of Cancer Research, UK) presented examples of slicing and dicing genetic data to identify the mechanistic basis of disease. Tatton-Brown selected patients with overgrowth phenotypes and found mutations in the histone linker gene *HIST1H1E*. Brunner took a directed approach by analyzing around 100 genes involved in the MTOR pathway and found a strong enrichment for increase in brain size, suggesting single or combinatorial effect of genes within this pathway cause microcephaly or macrocephaly phenotypes.

It is clear that the deluge of genetic data from sequencing cannot be relied upon for straightforward or intuition-based targeted subtyping of complex disease, but also will require brute force computational or high-throughput functional evaluations. Michael Johnson (Imperial College London, UK) demonstrated the strength of integrating genomic and phenotypic information with brain expression data to identify specific molecular networks disrupted in different types of epilepsy. Michael Brudno (University of Toronto, Canada) presented on a novel portal involving direct participation of patients to improve the quality of phenotyping—extending the depth of clinical data beyond that collected from a few minutes of interaction with the physician. Wyeth Wasserman (University of British Columbia, Canada) presented bioinformatics and deep learning methods that integrate experimental data, and structure-based and predictive models to infer active regulatory regions relevant to disease pathogenicity. Both tools show the growing importance in the field of using network and machine-learning-based approaches to find elusive connections between genes, pathways, and phenotypes. However, sometimes we simply need an innovative application of current molecular biology approaches to dissect the genetic architecture of a disease. Jay Shendure (University of Washington, USA) provided an exquisite example of going back to classic literature and revisiting the genetic cause for Lesch–Nyhan syndrome by generating CRISPR-mediated deletions tiling across the entire regulatory locus of the *HPRT1* gene. He convincingly showed that *HRPT1* gene expression is regulated by sequences proximal to the transcriptional start site near the first exon.

Model organisms provide us many insights into mechanisms and processes that are deeply conserved. Emma Farley (University of California, USA) showed an example using *Ciona intestinalis* to understand the order, orientation, and spacing properties of enhancer sequences and tissue-specific gene expression. Such assays are key to dissecting the function of regulatory elements in specific contexts of disease. The Lupski lecture on using organoids, given by Hans Clevers (Hubrecht Institute, The Netherlands), captivated the audience; it was promising to see the vast potential in modeling human organs and their pathologies for testing response to drugs, and editing for gene therapy. Specific examples of current forays in targeted therapy for genetic disorders were also presented. Notably, Frank Kooy (University of Antwerp, The Netherlands) provided an update on the promise of using ganaxolone, a modulator of the GABAergic system, for the treatment of fragile X syndrome and related neurodevelopmental disorders.

## Conclusions

Understanding which genetic variants to detect and how to interpret them, together with functional studies of molecular mechanisms, will pave the way for a clear understanding of the biological context of genetic variation in disease for better diagnoses and devising treatment modalities. This conference provided an update on current cutting-edge approaches used in studying the etiology of genetic disease. We are still cataloging variants and their function in physiology and disease variation will continue to be studied.

